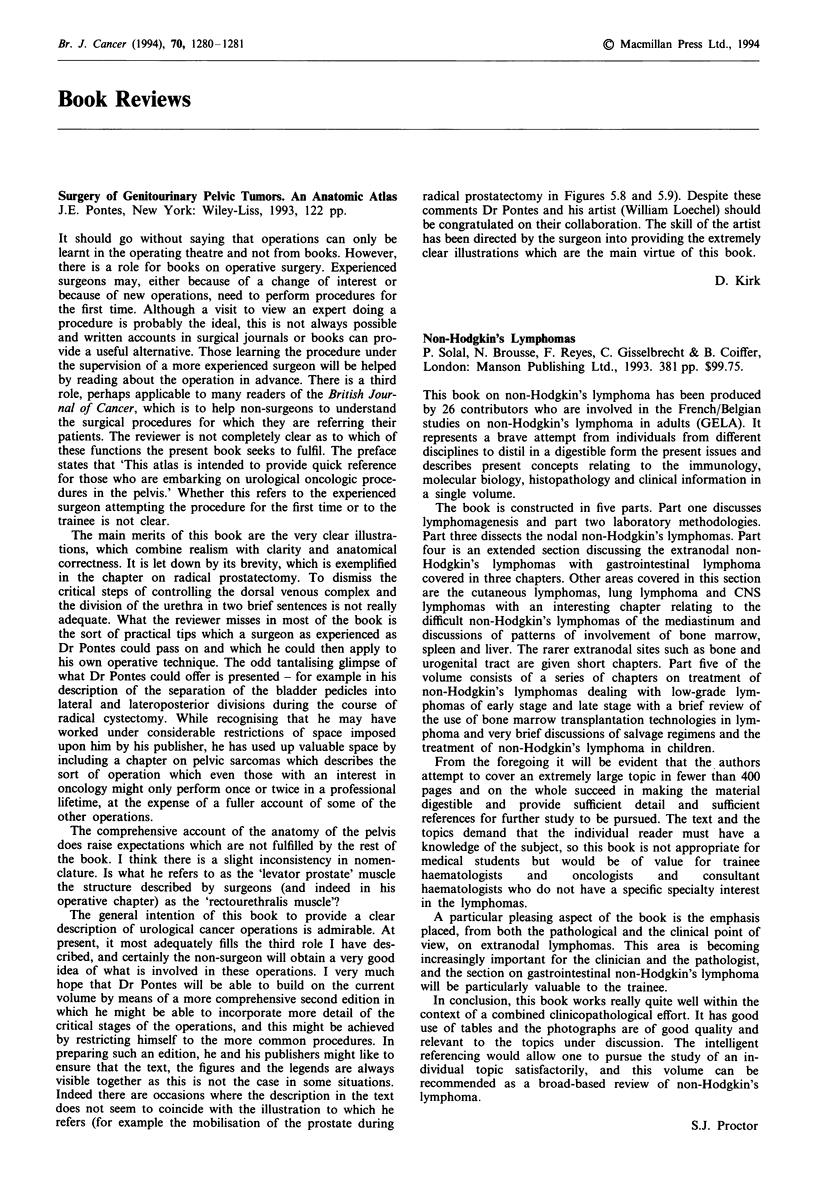# Surgery of Genitourinary Pelvic Tumors. An Anatomic Atlas

**Published:** 1994-12

**Authors:** D. Kirk


*Br. J. Cancer* (1994), **70**, 1280–1281 © Macmillan Press Ltd., 1994

### Book Reviews

**Surgery of Genitourinary Pelvic Tumors. An Anatomic Atlas**
J.E. Pontes, New York: Wiley-Liss, 1993, 122 pp.

It should go without saying that operations can only be learnt in the operating theatre and not from books. However, there is a role for books on operative surgery. Experienced surgeons may, either because of a change of interest or because of new operations, need to perform procedures for the first time. Although a visit to view an expert doing a procedure is probably the ideal, this is not always possible and written accounts in surgical journals or books can provide a useful alternative. Those learning the procedure under the supervision of a more experienced surgeon will be helped by reading about the operation in advance. There is a third role, perhaps applicable to many readers of the *British Journal of Cancer*, which is to help non-surgeons to understand the surgical procedures for which they are referring their patients. The reviewer is not completely clear as to which of these functions the present book seeks to fulfil. The preface states that 'This atlas is intended to provide quick reference for those who are embarking on urological oncologic procedures in the pelvis.' Whether this refers to the experienced surgeon attempting the procedure for the first time or to the trainee is not clear.

The main merits of this book are the very clear illustrations, which combine realism with clarity and anatomical correctness. It is let down by its brevity, which is exemplified in the chapter on radical prostatectomy. To dismiss the critical steps of controlling the dorsal venous complex and the division of the urethra in two brief sentences is not really adequate. What the reviewer misses in most of the book is the sort of practical tips which a surgeon as experienced as Dr Pontes could pass on and which he could then apply to his own operative technique. The odd tantalising glimpse of what Dr Pontes could offer is presented – for example in his description of the separation of the bladder pedicles into lateral and lateroposterior divisions during the course of radical cystectomy. While recognising that he may have worked under considerable restrictions of space imposed upon him by his publisher, he has used up valuable space by including a chapter on pelvic sarcomas which describes the sort of operation which even those with an interest in oncology might only perform once or twice in a professional lifetime, at the expense of a fuller account of some of the other operations.

The comprehensive account of the anatomy of the pelvis does raise expectations which are not fulfilled by the rest of the book. I think there is a slight inconsistency in nomenclature. Is what he refers to as the 'levator prostate' muscle the structure described by surgeons (and indeed in his operative chapter) as the 'rectourethralis muscle'?

The general intention of this book to provide a clear description of urological cancer operations is admirable. At present, it most adequately fills the third role I have described, and certainly the non-surgeon will obtain a very good idea of what is involved in these operations. I very much hope that Dr Pontes will be able to build on the current volume by means of a more comprehensive second edition in which he might be able to incorporate more detail of the critical stages of the operations, and this might be achieved by restricting himself to the more common procedures. In preparing such an edition, he and his publishers might like to ensure that the text, the figures and the legends are always visible together as this is not the case in some situations. Indeed there are occasions where the description in the text does not seem to coincide with the illustration to which he refers (for example the mobilisation of the prostate during radical prostatectomy in Figures 5.8 and 5.9). Despite these comments Dr Pontes and his artist (William Loechel) should be congratulated on their collaboration. The skill of the artist has been directed by the surgeon into providing the extremely clear illustrations which are the main virtue of this book.

D. Kirk